# A computerized automatic apparatus for determination of mercury in biological samples

**DOI:** 10.1155/S1463924684000158

**Published:** 1984

**Authors:** Östen Einarsson, Gösta Lindstedt, Torgil Bergström

**Affiliations:** National Board of Occupational Safety and Health Chemistry Division Ekelundsvägen 16 Solna S-171 84 Sweden


Journal of Automatic Chemistry, Volume 6, Number 2 (April–June 1984), pages 74–79

### A computerized automatic apparatus for determination of mercury in biological samples

**Östen Einarsson, Gösta Lindstedt and Torgil Bergström**

*National Board of Occupational Safety and Health, Chemistry Division, Ekelundsvägen 16, S-171 84 Solna, Sweden*

### Introduction

The determination of trace amounts of mercury in biological material is of considerable interest for the control of occupational exposure, as well as for the study of environmental pollution generally.

Atomic absorption analysis, which was introduced in about 1965, has become the most popular method for determination of trace metals. However, the standard technique for mercury is unfavourable because the sensitivity is not very high. Instead, a modification called 'flameless' (or cold vapour) atomic absorption has become the method of choice for determination of trace amounts of mercury. After the fundamental paper by Poluektov, Vitkun and Zelyukova [1] in 1964, hundreds of publications have described small variations of the same technique. $Hg^{2+}$ ions in solution are reduced by $Sn^{2+}$ or some other reducing agent to metallic mercury, which is driven out of the solution by an air or gas stream. The mercury-containing gas passes through a gas cell in a spectrophotometer, where its light absorption at 253·7 nm is measured.

In this laboratory, a rapid, flameless atomic absorption method for mercury in urine was worked out in 1970 by Lindstedt [2]. This principle is very well suited to automation and an instrument was later constructed by Lindstedt and Skare [3], which was able to analyse 60 digested urine or blood samples in about 2 h without supervision. The detection limit was about 1 ng of mercury per sample. This instrument worked well in the laboratory for more than 10 years; when it began to fail due to excessive wear, it was evident that a succeeding automatic mercury analyser ought to be computerized. Since the worn-out mercury analyser had functioned quite well, it was decided that the purgation-tower principle should be retained. Three separate pumps were introduced for transferring sample solution and tin solution to the tower and emptying the tower after the mercury had been blown out. The mechanical relays and switches were replaced with electronic tools.

Other automatic instruments for mercury analysis have been constructed by Agemian and Chau [4], Koirtyohann and Khalil [5], Matthes *et al.* [6], Coyle and Hartley [7] and others. Most of these instruments are based upon Technicon Auto-Analyzer equipment and different arrangements have been invented for the separation of the purging gas from the sample solution.

### Working principle of the apparatus

(1) Digested urine or blood samples (or other samples with mercury in solution) in plastic tubes are introduced into an automatic sample changer (60 samples).
(2) The first sample is pumped from the tube to a purgation tower, followed by a rinsing solution to check any traces of mercury solution from the tubing.
(3) A small volume of $Sn^{2+}$ solution is added to the solution in the tower.
(4) $Hg^{2+}$ in the sample is reduced to $Hg^0$ which is carried away from the tower by nitrogen which is introduced through the bottom of the tower.
(5) The mercury-containing gas passes through an absorption cell in an atomic absorption spectrophotometer; light absorption is read at 253·7 nm.
(6) When the light absorption has passed its peak and is decreasing, the tower is emptied and rinsed with water.
(7) When the light absorption has returned to its base-level (for example when there is no mercury left in the absorption cell), the sample changer moves one step forward and a new analysis is started.
(8) The light absorption is registered both by a recorder and by a printer. The mercury content of the samples is calculated by comparison with standard samples.

### Details of the apparatus (see figures 1 and 2)

*Sample changer*: Sampletron (Stålprodukter, Uppsala, Sweden), which takes 60 sample tubes (110 × 15 mm).

*Pumps*: three peristaltic pumps—Watson-Marlow H.R. flow inducer, type MHRE. Speed of pumping—$P_1$: 3·6 ml/s; $P_2$: 0·7 ml/s; $P_3$: 2·4 ml/s.

*Valves*: two magnetic valves—Burkert 114/A.

*Tubing*: silicone rubber, i.d. 4 mm (through the pumps) and Teflon, i.d. 2–3 mm (to the tower).

*Spectrophotometer*: Perkin-Elmer 306 with an EDL mercury lamp and a gas cell of plexiglass with quartz windows (l = 175 mm). The cell is shaped to follow the light beam as closely as possible in order to minimize dead space. Its section is rectangular and smallest at the middle (figure 1). The cell compartment is electrically heated to avoid condensed water in the cell.

*Recorder*: Perkin-Elmer Recorder 56.

*Rotameter*: Brooks Sho-Rate with a needle valve to regulate the nitrogen flow through the tower and cell.

*Tower*: a cylinder of Pyrex glass (length 175 mm, i.d. 17 mm) with a ground-glass joint, 19/26, at the top and a porous filter, P4, at the bottom. Below the filter, a three-way stopcock is fused to the cylinder to allow nitrogen to be introduced. The ground-glass stopper has a gas exit tube, and through the top, four Teflon tubings for sample, stannous chloride, rinsing liquid and draining are introduced.

*Computer*: Compucorp 445 Statistician programmable desk computer. This computer has a printer, which prints-out the maximum absorbance at the end of the working cycle.

*Control device*: a unit manufactured in the laboratory, containing seven identical circuits, which via a thyristor start or

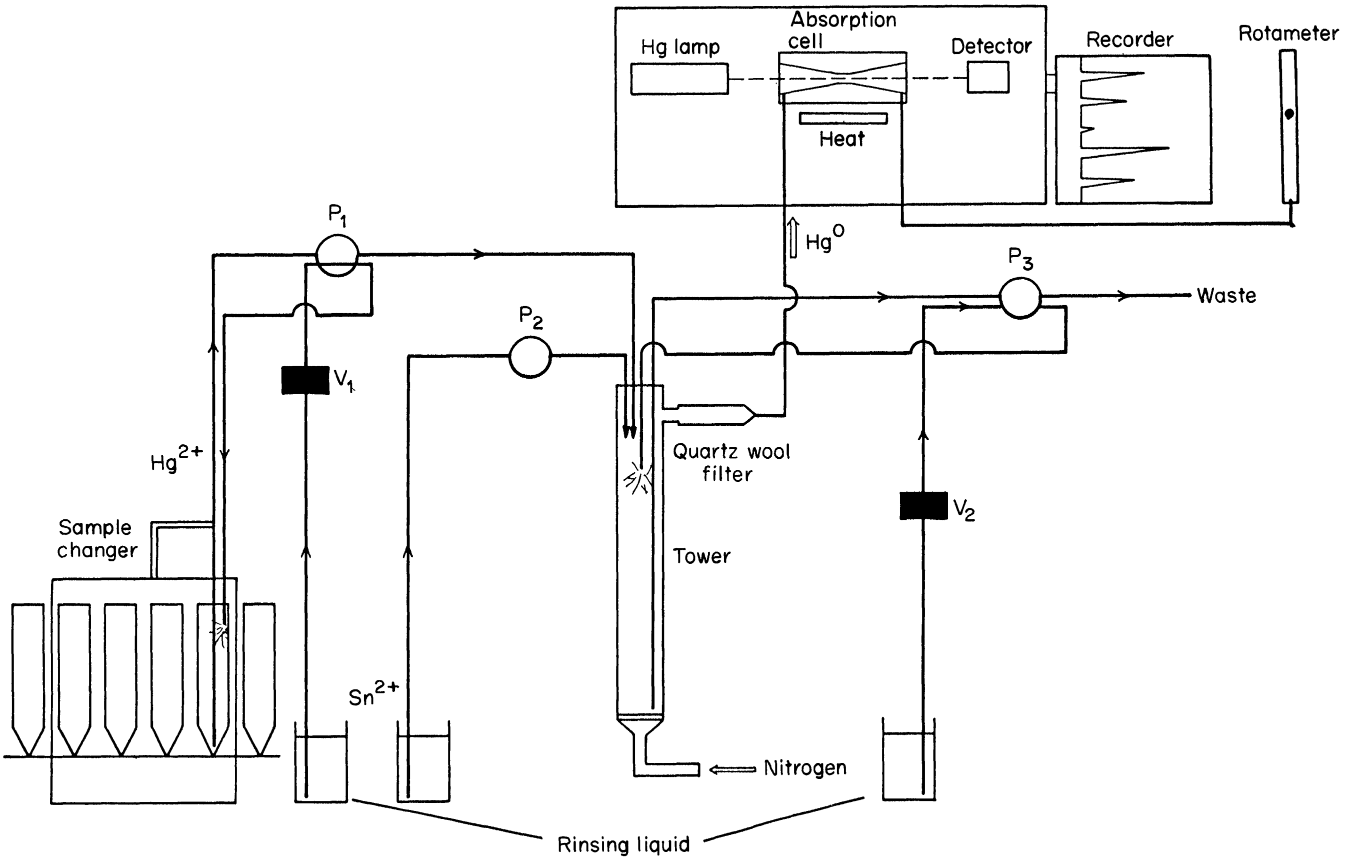

*Figure 1. Principal parts of the automatic mercury analyser. (Regulation mechanism excluded.)*

stop pumps, open or close magnetic valves and start the sample changer or the recorder paper when a signal is given from the interface. These signals can also be given manually. The control device contains a panel meter, on which the absorbance can be read digitally.

*Interface*: Datacollector DC-472, serving as an intermediate between the computer and the control device.

### Working cycle of the apparatus

The working cycle of the apparatus can be divided into 10 different phases (see table 1). The duration of the phases (except numbers 6 and 10) is regulated by an electronic clock in the DC-472, which is automatically zeroed at the beginning of the cycle. The length of each phase can easily be altered by changing the computer program.

Phase 6 is finished when the absorbance has reached its maximum and is decreasing. In practice, this phase lasts $40 \pm 2$ s. Phase 10 is finished when the absorbance has reached the base-level that it had in phase 2. After that the apparatus is at rest for another 30 s (phase 1), before the next analysis is started. The duration of phase 10 is 50–80 s, dependent on the amount of mercury in the sample. The whole working cycle takes 160–200 s. The flow of nitrogen through the tower and cell is continuous.

### Regulation mechanism (see figures 2 and 3)

The computer has both keyboard and machine instructions; the latter gives access to all 237 processor instructions. The program is stored on magnetic cards, which are stuck into a slot in the machine to load the program into the computer before each series of analyses is started.

The computer is connected to an interface (DC-472) with 72 inputs and 24 outputs and a built-in clock with a second counter (0–9999 s). The output of the interface has been modified with an inverting circuit (SN 7405) giving the inverted logic level and serving also as a buffer to protect the interface from interferences from relays etc. in the sample changer. The output signal to the control device is a low voltage (0–0·4 V) and no output signal is a high voltage (2·4–5 V). When the signal enters the control device it is inverted back to a normal logical voltage level. On the input to the inverting circuit (SN 7404) is a pull-up resistor which keeps the voltage high (= no signal) if the computer is not in use. In this case the control device can be regulated by hand, using push-buttons on the front panel. After the inverting circuit there is a line-driver circuit (SN 75454) giving signals to an opto-coupler (TIL 113), which transfers the signal to a thyristor (BT 138) which opens or closes the mains voltage to pumps and valves. The opto-coupler isolates the mains voltage part from the digital part, thus minimizing the risk of interference. At first, relays were used instead of thyristors, but these interfered with the electronics.

The analogue signal (absorbance) from the spectrophotometer passes through an operational amplifier and proceeds to a digital panel-meter, which shows the absorbance in digits on the display and also converts the figure to a digital number (BCD code). A cable from the panel-meter to the interface transfers these digital figures to the computer for further treatment. There is another cable from the ready-exit of the panel-meter to the interface. This cable receives a high voltage when a code is ready on the data output from the panel-meter. The computer then reads the digital BCD code via the interface. When this signal is low, the absorbance is being transformed and no data can be read.

*Table 1. Working scheme for different parts of the apparatus.*

| Part of apparatus | Phase No. 1 | 2 | 3 | 4 | 5 | 6 | 7 | 8 | 9 | 10 | Function |
|---|---|---|---|---|---|---|---|---|---|---|---|
| Pump $P_1$ | | | ■ | ■ | ■ | | ■ | ■ | | | Pumps sample from S to T and rinsing liquid into S and to T |
| Valve $V_1$ | | | | ■ | | ■* | ■ | | ■* | | Opens way to S for rinsing liquid |
| Pump $P_2$ | | | | ■ | | | | | | | Introduces $SnCl_2$ solution into T |
| Pump $P_3$ | | | | | | | ■ | ■ | ■ | | Drains T and pumps rinsing liquid into T |
| Valve $V_2$ | | | | | | | | | ■ | ■* | Opens way to T for rinsing liquid |
| Sample changer | | ■ | | | | | | | | | Moves turntable one step forward |
| Recorder | | ■ | ■ | ■ | ■ | ■ | | | | | Recorder chart moves |
| Atomic absorption spectrophotometer | | ■ | | | | | | | | ■ | Base-line absorption controlled before and after analysis |
| | | | | | | ■ | | | | | Peak absorption read |
| Printer | ■ | | | | | | | | | | Peak absorption of preceding sample printed |
| Duration of phase (s) | 30 | 5 | 3 | 1 | 4 | 40 | 4 | | 26 | 50–80 | |

■ = working, S = sample tube, T = Tower.

* The valves are opened for a few seconds to equilibrate pressure.

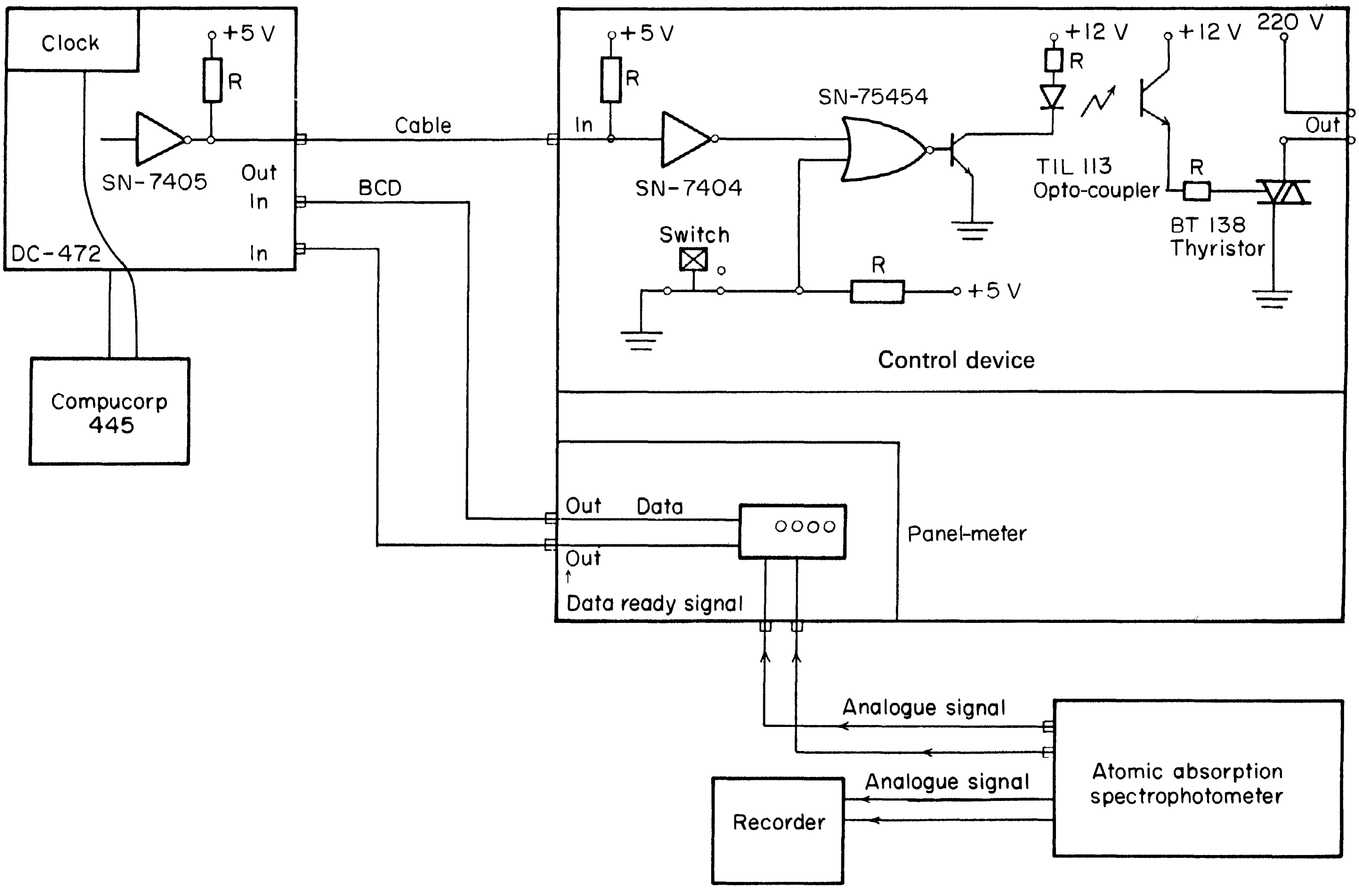

*Figure 2. Circuit diagram of the regulation mechanism.*

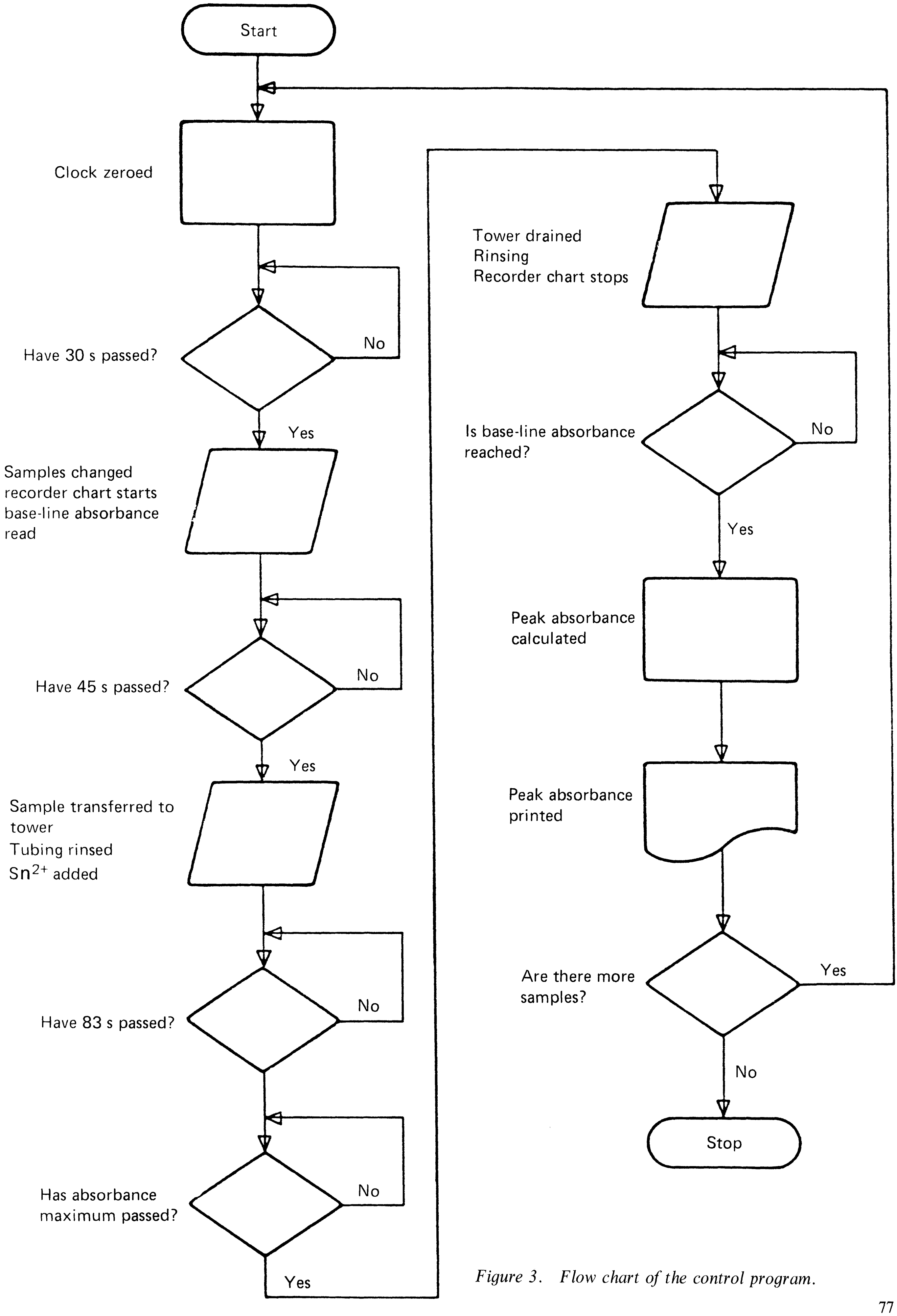

*Figure 3. Flow chart of the control program.*

### Procedure

*Samples*: urine samples (0·2 ml) in plastic tubes (110 × 15 mm) are digested by potassium permanganate-sulphuric acid overnight at room temperature as described by Lindstedt [2]. Next day, a 33% (w/v) solution of hydroxylamine hydrochloride in water is added drop by drop until the solution is colourless. The sample tube is then placed in the turntable of the sample changer. The total sample volume is about 2·2 ml.

Blood samples (0·2 ml) are digested with a nitric-perchloric acid mixture at 70°C overnight as described by Skare [8]; 1 ml of water and 125 μl of hydroxylamine hydrochloride solution are then added and the sample tubes placed in the turntable. Total volume is around 1·8 ml.

If it is possible, other biological samples are digested in the same way as blood samples. In some cases, special digestion methods have to be worked out.

Mercury in air is collected in personal sampling tubes containing manganese dioxide as described by Janssen *et al.* [9]. The adsorbent is dissolved in acid and a 2 ml aliquot is analysed in the automatic apparatus.

*Mercury standards*: each series of samples must be accompanied by some samples of the same type (urine, blood etc.) containing known amounts of mercury, which are analysed in the same way as the unknown samples. Urine or blood samples which are low in mercury are spiked with mercury standard solution corresponding to 0–100 μg Hg/l (=0–500 nmoles/l) for urine and 0–75 μg Hg/l (=0–380 nmoles/l) for blood.

*Instrument settings*: wavelength 253·7 nm, scale expansion 10X, recorder range 10 mV (for blood), 20 mV (for urine), nitrogen flow 100 ml/min.

*Analysis*: when the sample tubes are in the turntable of the sample changer, the atomic absorption spectrophotometer is switched on and allowed to reach equilibrium. The magnetic cards (three for the whole program) are stuck into the computer to introduce the program. The nitrogen flow is adjusted to 100 ml/min, the control device is switched on and a sample containing water is pumped into the tower by pressing the corresponding push-button. The tower is then drained manually. The series of analyses is then started by pressing the RESUME key on the computer. The apparatus now analyses the whole series of samples automatically and stops after the last sample.

*Calculation*: the light absorption of the samples is registered on the recorder strip and the peak absorption (in arbitrary units) is also printed on paper by the computer. The latter figures are generally used to set up the calibration graph from the standard samples, which can be done either manually or automatically by means of a desk computer steering an xy recorder. The calibration curve should be linear with good approximation within the range of mercury analysed. The mercury content of samples is calculated by comparison with the calibration graph. The absorption peaks on the recorder strip are used mainly as a control. Peaks of abnormal shape indicate some interference and such samples are analysed again.

### Results

### *Precision and detection limit*

Sixteen standard wate samples, each containing 10 ng of mercury, were analysed. The absorbances, expressed in printer units, were compared (mean:79·96 units; SD ±0·97; CV: ±1·22%; range: 77·8–81·5).

Twenty-five identical blood samples were analysed (mean: 133·25 nmol Hg/l; SD: ±4·34; CV: ±3·25%; range: 127·1–141·4).

If the limit of detection is defined as twice the standard deviation, it will be 0·25 ng Hg/sample for aqueous standard and 0·34 ng Hg/sample for blood. Since each blood sample contains 0·2 ml of blood, the lowest detectable mercury content in blood will be 1·7 ng/ml or 8·5 nmol/l. The normal blood mercury in occupationally unexposed persons is generally higher (4–5 ng/ml).

### *Different methods of evaluation*

The mercury content of the samples can be calculated from their light absorption in three different ways: (1) from the printer figures; (2) by measuring the peak heights on the recorder strip; (3) by measuring the peak areas. In more sophisticated instruments, the integration of peak area is performed electronically. Since this facility was not available, the printed peak areas were measured by weighing after cutting them out with scissors. The integration method requires the computer program to be changed to allow all mercury to be aerated out of the tower and cell before the tower is drained of solution. It is also essential that the nitrogen flow is kept constant during the whole series of analyses. The peak height is less sensitive to small changes in gas flow [2].

Six different urine samples were analysed (five parallel runs of each), and the absorption evaluated by all three models. Between the printer figures and peak heights, there was always a constant factor of 1·20±0·01. These figures both reflect maximum absorbance, one measured manually and the other electronically. The quotient between paper weight (mg) and peak height is subject to a larger variation: from 1·05 to 1·20.

Within each series of parallel runs, the variation was lowest for the peak height method (CV 1·4–3·8%) and highest for the peak area method (CV 1·5–6·3%).

### *Variation in sensitivity for different matrices*

As indicated above, the mercury content of the samples analysed is determined by comparison with standard samples prepared from a blood or urine originally containing very little mercury. It is then assumed that the sensitivity (the slope of the calibration curve) is the same for all urine or blood samples. If not, the only alternative would be to make standard additions to each sample analysed. The latter method is often used in atomic absorption analysis but it is time-consuming since the number of samples which need to be analysed is more than double compared to the use of calibration curves.

To discover variation in sensitivity for different matrices, three urine samples were spiked with mercury standard corresponding to 250 nmol/l and the increase in absorbance studied. The latter was estimated both by peak height and peak area. Five parallel runs were made on each urine.

For peak height, the increase was 62·6±2·5, 69·1±1·1 and 64·7±1·6 scale units, respectively. The corresponding figures for peak area were 75·6±2·5, 74·8±1·3 and 74·8±2·0 mg paper weight. (The close resemblance between the figures for peak height and paper weight is purely coincidental.)

For peak height, the second sample differs significantly ($p<0·001$) from the two others, indicating that sensitivity is dependent on the matrix, for example unknown differences in composition of the urines. If the second sample is used as standard for evaluation of the first sample, an error of 10% would be made.

However, the peak area differs by only 1% between the three urine samples, this difference is not significant.

Since blood mercury is more important for occupational exposure control, a more extensive investigation was made on blood samples. Nine different samples low in mercury were spiked with mercury standard corresponding to 374 nmol/l. All samples were analysed in triplicate both before and after the addition of mercury standard. The increase in absorption was estimated by peak height and by peak area.

For peak height, the average increase was 77·9 ± 2·2 scale units (range 72·3–79·6) and for peak area, 65·7 ± 1·6 mg paper (range 62·2–67·5). The corresponding coefficients of variation are rather similar: 2·90% and 2·39% respectively. No direct conclusions can be drawn from these figures about which method is most independent of the sample matrix. Analysis of variance, however, showed that the difference in absorbance increase between samples is significant for the peak height ($p = 0{\cdot}01$) and not significant ($0{\cdot}05 < p < 0{\cdot}2$) for peak area. So peak area is a safer method of evaluation than peak height. In practice, however, if the samples are analysed only for estimation of occupational exposure, an error of 10% can be tolerated and the less complicated peak height method justifiably used for routine purposes.

### *Variation in sensitivity during one year's use*

As shown above, within-day variation in sensitivity may be related to differences in matrix composition between different samples. Between-day variation may also be influenced by varying purity in the porous filter in the tower, as well as by small differences in detector gain setting from day to day. If some pores of the filter are clogged, the gas bubbles generally get fewer and bigger, thus causing less effective purging of the solution. The peaks are then flat and extended, and the sensitivity is decreased.

As the instrument has been in routine use for more than a year, there is sufficient material to study long-term variation in sensitivity.

The mercury standard samples added to each series of analyses are well suited to make an estimation of the variation in sensitivity. The increase in absorbance for the highest standard sample was studied for urine and blood for all analyses performed during 1982.

For urine, the increase in absorbance for an addition of 499 nmol Hg/l was 131·4 ± 8·7 printer units ($N = 37$, CV = 6·60%; range: 117·2–155·7). In absolute units, the average sensitivity is calculated to $4{\cdot}4 \times 10^{-4}$ absorbance units/nmol/l.

For blood, an addition of 374 nmol Hg/l gave an average increase in absorbance corresponding to 76·6 ± 5·3 printer units ($N = 54$, CV = 6·93%; range: 66·9–89·1). The average sensitivity is $3{\cdot}4 \times 10^{-4}$ absorbance units/nmol/l.

As might be expected, variation over a year is greater than within one series of analyses. It is remarkable that the sensitivity for blood is more than 20% lower than for urine. The reason could be that the blood samples are more viscous even after digestion, giving a less even distribution of gas bubbles than urine samples. Another contributing factor may be the loss of mercury by evaporation during the digestion of blood. But control experiments have shown that only 6–7% of the mercury is lost if the digestion is carried out overnight at 70°C. The accuracy of the analysis is not influenced by that loss, since the mercury standards are treated in the same way as the samples to be analysed.

### Conclusions

The apparatus described here makes a rapid and convenient analysis of mercury in urine and blood samples, as well as in aqueous solutions. The sensitivity is high enough for determination of mercury samples from occupationally unexposed persons. The variations in sensitivity due to matrix influences are within 10%, which is acceptable for routine control.

The matrix influence could be considerably reduced if the peak area method were used for evaluation of the absorbance rather than the peak height method. It would be possible to do this with by some changes to the computer program, but some other precautions would also have to be taken to keep the gas flow constant and the speed of analysis would then be lowered.

Some authors have recommended other reducing agents, such as alkaline tin (stannite) solution [10] or sodium borohydride [11]. Both reagents allow mercury in urine or blood to be determined without having to digest the sample and neither of them is affected by bromide or iodide ions, unlike acid tin solution. Furthermore, the stannite reduction method is able to distinguish between $Hg^{2+}$ and organic mercury, and sodium borohydride gives a faster reduction than tin solution. However, these reagents were not tested because alkaline solutions would cause problems as a result of heavy foaming in the purgation tower.

### Acknowledgements

The authors are indebted to Mrs K. Roxström, S. Siljerud and B. Åkerlund for the analytical work, to Mrs K. Oxelbeck for typing the manuscript and to Miss K. Williams, B.Sc., for revising the English.

### References

1. Poluektov, N. S., Vitkun, R. A. and Zelyukova, Yu., V., *Zhurnal Analiticheskoii Khimii*, **19** (1964), 937.
2. Lindstedt, G., *Analyst*, **95** (1970), 264.
3. Lindstedt, G. and Skare, I., *Analyst*, **96** (1971), 223.
4. Agemian, H. and Chau, A. S. Y., *Analytica Chimica Acta*, **75** (1975), 297.
5. Koirtyohann, S. R. and Khalil, M., *Analytical Chemistry*, **48** (1976), 136.
6 Matthes, W., Flucht, R. and Stoeppler, M., *Fresenius' Zeitschrift für Analytische Chemie*, **291** (1978), 20.
7. Coyle, P. and Hartley, T., *Analytical Chemistry*, **53** (1981), 354.
8. Skare, I., *Analyst*, **97** (1972), 148.
9. Janssen, J. H., van den Enk, J. E., Bult, R. and de Groot, D. C., *Analytica Chimica Acta*, **92** (1977), 71.
10. Magos, L., *Analyst*, **96** (1971), 847.
11. Sharma, D. C. and Davis, P. S., *Clinical Chemistry*, **25** (1979), 769.

### EASTERN ANALYTICAL SYMPOSIUM

*13–16 November 1984 in New York, USA*

The 22nd EAS held in November last year attracted over 4500 participants (33% more than the 1982 meeting) so this year's conference will run over four days rather than three. The symposium will once again be at the New York Penta Hotel in New York City. Technical sessions will be held on all four days, permitting an expansion to 45 sessions; in the EAS tradition, these will consist principally of invited papers. Based upon the success of the poster sessions held at the 1983 EAS, these will also be expanded for 1984, and will include invited as well as contributed posters. The exhibit area of the accompanying exposition has been increased to accomodate a total of around 190 booths. Sponsoring organizations include the American Microchemical Society, the American Chemical Society and the Society for Applied Spectroscopy.

*Additional information from Merck & Co. Inc., PO Box 2000, Rahway, New Jersey 07065, USA.*

